# Immunogenicity of an Inactivated DIVA Lumpy Skin Disease Virus Vaccine in Guinea Pigs and Lactating Cows, and Its Effects on Cow Lactation

**DOI:** 10.3390/vaccines14050370

**Published:** 2026-04-22

**Authors:** Lilia Testa, Sara Capista, Anna Serroni, Mariangela Iorio, Gaetano Federico Ronchi, Sara Traini, Ivano Di Matteo, Caterina Laguardia, Francesca Profeta, Cristiano Palucci, Marco Caporale, Maria Antonietta Saletti, Alice Marchegiano, Chiara Pinoni, Emanuela Rossi, Romolo Salini, Graziano Aretusi, Gisella Armillotta, Sara Fanì, Francesca Parolini, Mauro Di Ventura, Maria Teresa Mercante

**Affiliations:** 1 Istituto Zooprofilattico Sperimentale dell’Abruzzo e del Molise G. Caporale, 64100 Teramo, Italy; l.testa@izs.it (L.T.); s.capista@izs.it (S.C.); m.iorio@izs.it (M.I.); f.ronchi@izs.it (G.F.R.); s.traini@izs.it (S.T.); i.dimatteo@izs.it (I.D.M.); c.laguardia@izs.it (C.L.); f.profeta@izs.it (F.P.); c.palucci@izs.it (C.P.); m.caporale@izs.it (M.C.); m.saletti@izs.it (M.A.S.); a.marchegiano@izs.it (A.M.); c.pinoni@izs.it (C.P.); e.rossi@izs.it (E.R.); r.salini@izs.it (R.S.); g.aretusi@izs.it (G.A.); g.armillotta@izs.it (G.A.); s.fani@izs.it (S.F.); f.parolini@izs.it (F.P.); mauro.diventura@gmail.com (M.D.V.); t.mercante@izs.it (M.T.M.); 2 Department of Veterinary Medicine, University of Teramo, 64100 Teramo, Italy

**Keywords:** lumpy skin disease, vaccine, DIVA, immunogenicity, guinea pigs, lactating cows

## Abstract

Background: Lumpy skin disease (LSD) is caused by a *Capripoxvirus*. Live attenuated vaccines, which are commercially available, could be not safe because of the side effects. The aim of this study was the evaluation of the safety, immunogenicity, and effects on the qualitative and quantitative parameters of milk. The feasibility of identifying vaccinated animals using our inactivated vaccine in dairy cows was analysed. The vaccine was tested in guinea pigs as an immunogenicity predictive model. Methods: LSD virus was propagated on Madin–Darby Bovine Kidney (MDBK) cells, then inactivated and supplemented with keyhole limpet hemocyanin (KLH) protein, obtaining a positive marker vaccine. This was inoculated in guinea pigs and in dairy cows, and animal sera were analysed using enzyme-linked immunosorbent assay (ELISA) and a serum neutralisation (SN) test. Quantitative and qualitative analyses were performed on milk. Results: The vaccine was previously tested for efficacy in vaccinated calves, showing a pronounced reduction in clinical symptoms after challenge. The safety and immunogenicity obtained in calves were also confirmed in dairy cows in this study. In fact, high values of the SN test (1:20 to 1:80) and ELISA (90 and 240 S/P%) were obtained after vaccination. Moreover, high immunogenicity of the vaccine was also assessed in guinea pigs. In addition, the results of the milk analyses did not show any differences between vaccinated and control groups. The KLH was able to elicit an immune response detectable using an ELISA (3.0 and 3.5 optical density values). Finally, our vaccine could be used to reduce LSD symptoms and identify vaccinated animals.

## 1. Introduction

Lumpy skin disease (LSD) is a viral disease that naturally affects cattle, especially the young and cows at the peak of lactation [1,2]. The lumpy skin disease virus is the aetiological agent of LSD and belongs to the *Poxviridae* family and *Capripoxvirus* genus. The transmission is primarily due to hematophagous biting midges [3], which act as mechanical vectors rather than biological ones [4,5].

Lumpy skin disease is usually characterised by a low mortality rate (up to 10%) and is responsible for different clinical signs in infected animals that may vary from mild subclinical forms to more severe symptoms, such as ocular and nasal discharge, high fever, and multiple nodular skin lesions with subsequent poor skin quality. Moreover, typical LSD symptoms include abortion, temporary or permanent infertility, and a notable decrease in milk production [6], leading to huge economic losses for the cattle industry [2].

Lumpy skin disease was first detected in 1929 in Zambia, and rapidly became endemic in most African countries [1,6,7]. Since 2012, many LSD outbreaks have been reported in Middle East regions. The first LSD incursion occurred in south-eastern Europe in 2015–2016 (Greece, Bulgaria, Serbia, Albania and Montenegro) [7,8].

As of June 2025, LSD was detected for the first time in Italy, where 80 outbreaks have occurred [9], mostly exclusively located in Sardinia region, Nuoro province. Only one outbreak was detected in mainland Italy (Lombardy region, Mantua province), which was the consequence of the legal movement of animals from the first confirmed outbreak in Sardinia. Phylogenetic analyses have also been conducted by the European Union Reference Laboratory (EURL), and the results suggested a genetic link between the LSD virus detected in Italy and the Nigeria 2018 strain [10].

In order to contain the outbreaks, Italy planned an emergency protective vaccination in accordance with Regulation (EU) 2023/361. The strategy targets 300,000 domestic bovines across the entire Sardinia region using the live attenuated OBP Neethling strain vaccine (Onderstepoort Biological Products, Pretoria, South Africa). Recently, LSD outbreaks were also confirmed in Catalunya (Spain), near the French border [11].

Prophylaxis is performed with the only commercially available attenuated vaccine. It may cause clinical signs similar to those observed in cases of natural infection by LSDV, such as fever, viraemia, decreased feed intake, variable-sized cutaneous nodules everywhere on the body, and finally decreased milk production, which between the reported side effects of attenuated LSD vaccines is one of the most representative adverse reactions [12]. The use of these vaccines in LSD-free countries has some drawbacks, since it involves circulation of the virus, even if attenuated, and could cause “Neethling disease”. Therefore, since there is a concrete risk of LSD spreading from Italy to other European countries, it would be more advisable to use an inactivated, safe and effective vaccine that does not affect milk production, avoiding further huge economic losses.

Inactivated vaccines against LSD have already been produced [13,14], but none of them have been tested in lactating cows. Therefore, previous studies are lacking scientific data on milk production in LSD-vaccinated animals. Moreover, the inactivated vaccines already produced were lacking a marker that could help to identify vaccinated animals using an ELISA. The introduction of a positive marker in a vaccine formulation, together with a negative marker (deleted vaccine), would be the best option for applying the DIVA strategy. The DIVA vaccine could be produced with a deletion (negative marker) or adding an external protein (positive marker). Marker vaccines are designed for conferring protection and for distinguishing infected from vaccinated animals. The DIVA strategy is the most useful tool for eradicating infectious diseases of domestic animals in different countries. In our laboratory, an inactivated vaccine was previously produced, and it was formulated with the addition of the commercial exogenous protein KLH, which is extracted from a crustacean (*Megathura crenulata*), as a positive marker. The vaccinated animals developed an immune response against the LSD viral antigen and KLH. The KLH immune response allows for the identification of vaccinated animals through a specific ELISA [15,16,17]. This last property could be useful in vaccination campaigns where the use of a unique vaccine is often mandatory, enabling authorities to identify vaccinated animals and assess compliance with vaccination programmes.

The aim of this study was the evaluation of safety, immunogenicity, and effects on lactation of our developed positive marker inactivated vaccine against lumpy skin disease in lactating cows, and the evaluation of suitability of guinea pig as animal model to be used for predicting the immunogenicity of this vaccine.

## 2. Materials and Methods

### 2.1. Inactivated Vaccine Production

The detailed procedures for the preparation of the inactivated LSD vaccine that was used in this study is described by Ronchi et al. [15]. Briefly, the vaccine was produced from a field viral strain isolated from a symptomatic calfskin nodule, as described by Babiuk et al. [18], that was collected during an outbreak in Albania in 2017, and was kindly provided by the Food Safety and Veterinary Institute (FSVI) in Tirana. The primary viral stock was obtained through consecutive viral amplification passages on the Ovine Aries Testis cell line (OA3.Ts cells -ATCC Cat# CRL-6546, RRID: CVCL_3764), a continuous cell line susceptible to LSD virus infection, and it was checked for sterility for bacteria, fungi, and mycoplasma and for viral identity and titre as prescribed in the European Pharmacopoeia [19].

The vaccine was then prepared through infection of Madin–Darby bovine kidney (MDBK) cells (ATCC Cat# CRL-6071, RRID: CVCL_0421) with the above-mentioned field viral strain. The cells were cultured in minimum essential medium (MEM, Gibco, Waltham, MA, USA) at 37 °C and 5% CO_2_.

The viral suspension was concentrated through tangential flow filtration using a Cogent M1 trans-membrane pressure (TMP) system (Merck Millipore, Burlington, MA, USA), and then subjected to chemical inactivation with a 5 mM binary ethylenimine (BEI) solution, performed according to Bahnemann [20].

The viral suspension, after performing an inactivation control as Ronchi et al. described [15], was adjuvanted with 10% (*v*/*v*) Montanide Gel 01 PR (provided by Seppic, Paris, France) and was supplemented with 0.03 mg/mL saponin (Sigma, Cat# SAE0073, St. Louis, MO, USA) and 0.13 mg/mL of KLH to fulfil a DIVA vaccinology strategy purpose. The sterility for bacteria, fungi and mycoplasma was also verified according to the European Pharmacopoeia [19] in this final vaccine formulation, before using it in the animal experimentation.

The placebo formulation used for inoculating the guinea pigs in successive experiments was composed of phosphate-buffered saline (PBS), KLH, Montanide Gel, and saponin in the same concentrations used in the vaccine formulation.

### 2.2. Ethics Statement

The animal study protocol was reviewed and authorised by the Italian Ministry of Health (authorization no. 517/2021-PR of 12 July 2021). According to the public sanitary authorization code 011TE286, the study on the cows was carried out at the originating farm located in Castellalto, Teramo (Italy), and for this reason the animals did not require an acclimation period in a new environment. The study involving the guinea pigs was conducted within the sealed facility 041/TE629, BDF16 (authorization No. 79/2014-A, issued on 28/02/2014, in compliance with Legislative Decree No. 116/1992).

### 2.3. Animal Experimentation

In order to define any potential side effect of this vaccine on lactation, the vaccine was firstly tested on 20 female Friesian lactating cows aged between 2 and 11 years chosen for experimentation. Ten animals received two vaccine doses (2 mL) intramuscularly, i.e., one dose at day 0 and a booster dose at day 28 of the experimentation. The other 10 animals represented the negative control group, which did not receive any treatment and/or inoculation. The cows were subjected to a physical examination daily, evaluating the environment, behaviour and social interaction in addition to the general physical condition, which entailed inspection of the injection site. Moreover, animal body temperatures were monitored daily starting 14 days before the first vaccination, with monitoring 4 h after the vaccine administration and 14 days consecutively after immunisation. The same timing was applied to check the temperature after the booster dose (day 28). A temperature increase above 40 °C was considered indicative of fever. The trial on lactation cows lasted 63 days.

The vaccine was then also tested in Dunkin Hartley guinea pigs: the animals weighed 400 g, and were males and females. The experimentation lasted 60 days, and included 20 guinea pigs, 12 of which were inoculated with 1 mL vaccine by intramuscular route, and the remaining 8 animals were kept as negative control and were inoculated with the placebo formulated as above reported. A booster dose was administered 28 days after the first one.

### 2.4. Serological Analyses

Whole-blood samples were taken from lactating cows every week for two months consecutively, starting from the first day of vaccination to day 63, to assess the immune response against LSD virus including γ-interferon production. In addition, guinea pigs were sampled on days 0, 28 and 60 to evaluate the immune response until 60 days. The IgM and IgG responses against the exogenous protein KLH were only analysed in the vaccinated lactating cows, as the negative control did not receive any treatment.

#### 2.4.1. ELISA and SN

The immune response of animals against the virus was detected using enzyme-linked immunosorbent assay (ELISA). Serum samples were analysed using an ID Screen Capripox Double Antigen Multi-species ELISA (Innovative Diagnostics, Montpellier, France, Cat#CPVDA). The test was performed according to the manufacturer’s instructions and the samples with an S/P% ratio value ≥ 30% were considered positive.

The serum neutralisation test was performed as described by Ronchi et al. [15], following the Terrestrial Animal Health Code of the World Organization for Animal Health [3].

#### 2.4.2. Interferon-γ Assay

The interferon-γ (IFN-γ) ELISA was performed using a commercial ELISA PRO: Bovine IFN-γ kit (Mabtech, Stockholm, Sweden, Cat#3119), according to the manufacturer’s protocol as described by Ronchi et al. [15]. All samples and standards were analysed in duplicate.

#### 2.4.3. ELISA for KLH Antibodies

For quantifying the anti-KLH antibodies, the commercial Bovine Anti-Keyhole Limpet Hemocyanin (KLH) IgM ELISA Kit and Bovine Anti Keyhole Limpet Hemocyanin (KLH) IgG ELISA Kit (Alpha Diagnostic International, San Antonio, TX, USA, Cat# 700–165-KBM and Cat# 700–160-KBG, respectively) were used according to the manufacturer’s protocols, including the interpretation of results.

### 2.5. Study on Lactation

Milk samples were collected daily from the animals of the two experimental groups, starting from the first day of vaccination (both first and second dose) and for 14 days consecutively after immunisation, in order to evaluate the quantity of produced milk and the chemical–physical parameters (casein, somatic cells, lipids, proteins and dry fat residue) using infrared spectroscopy.

Samples were processed at the Food Hygiene and Technologies Laboratory of Istituto Zooprofilattico Sperimentale Abruzzo and Molise “G. Caporale” in Lanciano (Italy).

### 2.6. Statistical Analyses

Statistical analyses were performed to compare the immune response, release of IFN-γ, and milk production between the vaccinated and control group, applying a non-parametric Mann–Whitney test. All tests were conducted using Microsoft Excel 2016 (Microsoft Corporation, Washington, DC, USA), R version 4.4.2 (Core Team, Jaipure, RAJ, India), and RStudio Posit team (2025), RStudio: Integrated Development Environment for R (version 2025.09.0, “Cucumberleaf Sunflower” Posit Software, PBC, Boston, MA, USA).

## 3. Results

### 3.1. Inactivated Vaccine Production

The field virus used for vaccine production, isolated from a biopsy and propagated in OA3.Ts cells, had a titre of 10^5.3^ tissue culture infectious dose 50% (TCID_50_/mL). The titre of the virus used for vaccine production, before concentration and inactivation, was equal to 10^5.6^ TCID_50_/mL. Identity and purity tests produced favourable results, and the inactivation controls confirmed the absence of virus replication. Additionally, the formulation was proven sterile, showing no contamination by bacteria, fungi, or mycoplasma.

### 3.2. Inoculation of Animals

No adverse reactions were observed following the administration of the vaccine formulation; only one lactating cow showed a reaction at the injection site, which then regressed spontaneously.

**Figure 1:**
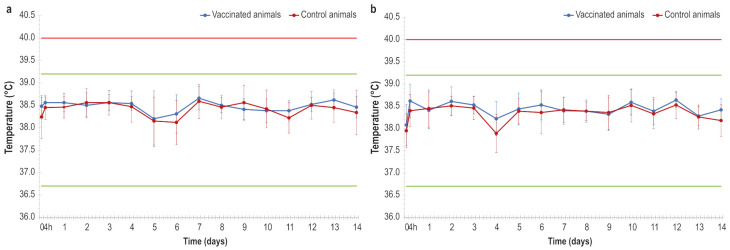
(**a**) Mean animal temperatures detected in vaccinated and control animals after receiving the first vaccine dose and placebo, 4 h post injection, and the following 14 days. (**b**) Mean animal temperatures detected in vaccinated and control animals after receiving the second vaccine dose and placebo, 4 h post injection, and the following 14 days. Red line: threshold above which the temperature is considered fever. Green lines: the highest and lowest temperatures detected in lactating cows before the vaccination. Error bars: standard deviations.

There were no significant increases in body temperature throughout the trial (Figure 1a,b).

### 3.3. Immunological Assays

#### 3.3.1. ELISA and SN Test in Lactating Cows

To evaluate the vaccine immunogenicity, serum samples were analysed using a commercial ELISA kit, as previously described.

**Figure 2:**
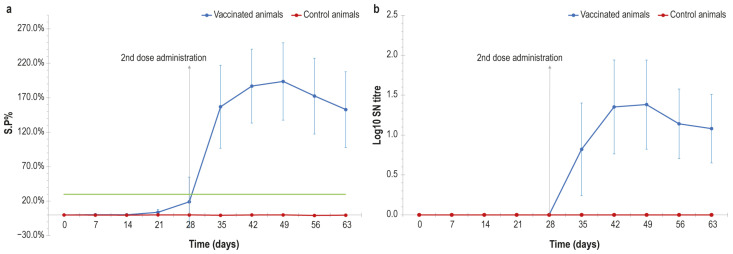
(**a**) S/P values (%) obtained in ELISA from serum samples of lactating cows (vaccinated and control group), represented over time (days). Green line: cut-off. Samples with S/P% above 30% are considered positive. (**b**) Graphical representation of LSDV-neutralising-antibody titres analysed from vaccinated and control lactating cows, expressed in log_10_ over time. Error bars: standard deviations.

Regarding the experiment in lactating cows, after the first vaccine administration (day 0) antibody titres of both animal groups (vaccinated and control) were below the cut-off (30% S/P%) until day 28, as shown in Figure 2a.

A week after the booster dose that the animals received at day 28, an increase in titre values was observed in the vaccinated group. The antibody titres raised to their highest value at day 49, when the animals were all positive, with titres ranging from 90 to 242 S/P%. The ELISA positivity lasted above the cut-off level until the last day of experimentation (day 63). The control group did not show any variation in the antibody titres over time. The statistical analysis showed a significant difference (*p* value = 0.0018) between the two groups (vaccinated and control) starting at day 28, thus suggesting an effective immunological response in vaccinated animals.

The immunological response of vaccinated animals was also investigated using serum neutralisation tests. The results trend was similar as that obtained in ELISA; indeed, all the lactating cows were negative until day 28. After the booster dose the seroconversion in vaccinated animals started, with titre values ranging from 1:10 to 1:80 until day 63 (Figure 2b) and showing the highest value at day 49. At this time point, all the animals were positive, with titres ranging from 1:10 to 1:80. The mean values of log_10_ SN titres were >0 in vaccinated cows from day 28 until the last day of experimentation; conversely, control animals were negative from the first to the last day. This difference was statistically significant (*p* value < 0.001) at 42 days.

#### 3.3.2. ELISA and SN Test in Guinea Pigs

**Figure 3:**
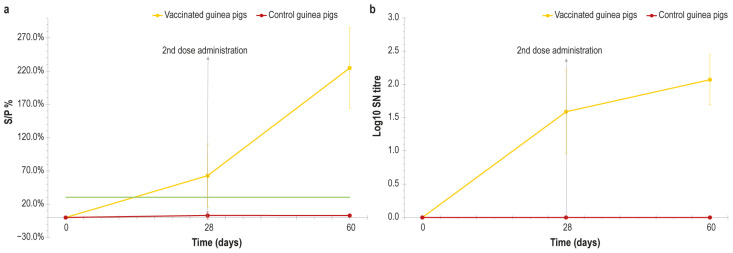
(**a**) S/P values (%) obtained in ELISA from serum samples from guinea pigs (vaccinated and control group), represented over time (days). Green line: cut-off. Samples with S/P% above 30% are considered positive. (**b**) Graphical representation of LSDV-neutralising-antibody titres analysed from the vaccinated and control group, expressed in log_10_ over time. Error bars: standard deviations.

The ELISA performed in vaccinated guinea pig samples revealed that the immunological response following vaccination was obtained in the days immediately after the inoculation, reaching the highest value at day 60, the last day of experimentation (Figure 3a). The control animals were negative from the first day until the end of the experiment, and the statistical analysis showed that the difference between the vaccinated and control group was significant, with *p* values equal to 0.01 and 0.0018 at 28 and 60 days respectively.

The rapid immunological response detected in ELISA was confirmed through a serum neutralisation test in guinea pigs. Indeed, as shown in Figure 3b, the SN titre rapidly increased in vaccinated animals, even before the second vaccine administration at day 28, and reached the highest value at day 60. Control animals were negative for the entire duration of the experiment, and the difference between the two groups was statistically significant with *p* values ≤ 0.001 at 28 and 60 days.

#### 3.3.3. Interferon-γ Assay

**Figure 4:**
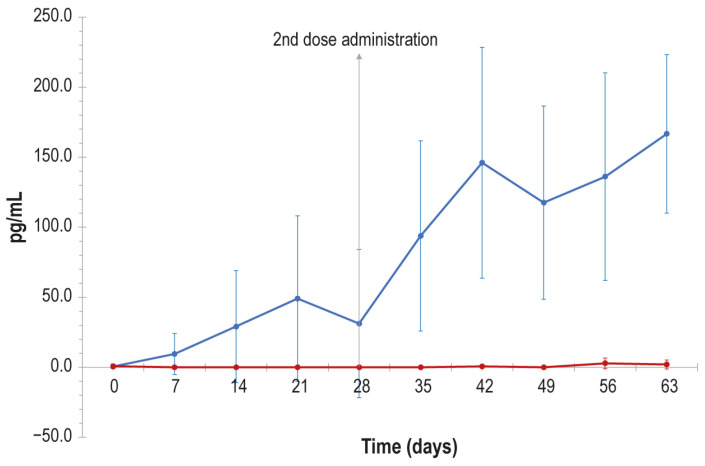
Interferon-γ concentration (pg/mL) detected in blood samples of lactating cows in the vaccinated and control group over time (days). Blue line: vaccinated animals; red line control animals. Error bars: standard deviations.

All samples from vaccinated lactating cows subjected to in vitro stimulation with inactivated LSDV were found to be positive for interferon-γ production, in contrast to the control animal samples which did not show any increase in interferon-γ concentration after stimulation (Figure 4).

In particular, a substantial increase in interferon-γ concentration was detected 7 days after the first vaccine administration. A slight reduction in interferon-γ was appreciable at day 28, when the second dose of vaccine was injected. After the booster dose a further interferon-γ increase was detected, which reached its highest value at day 63. The difference in interferon-γ concentration between the vaccinated and the control group was statistically significant starting from day 7 (*p* value < 0.05).

#### 3.3.4. ELISA for KLH Antibodies

**Figure 5:**
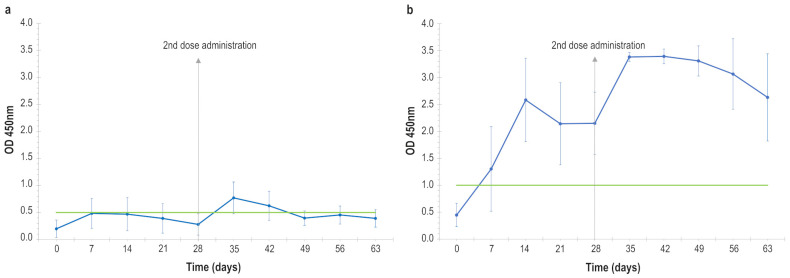
(**a**) Optical density values obtained by ELISA for anti-KLH IgG detection from serum samples of vaccinated lactating cows, represented over time (days). (**b**) Optical density values obtained by ELISA for anti-KLH IgM detection from serum samples of vaccinated lactating cows, represented over time (days). The green line represents the cut-off above which the samples are considered positive. Error bars: standard deviations.

The vaccine was supplemented with the exogenous protein KLH in order to distinguish vaccinated animals from unvaccinated ones. For this purpose, an immunoassay able to detect anti-KLH IgM and IgG antibodies was performed. According to the results, the IgM antibody titre was negative at all the experimental time points except for day 35, with a maximum value of 1.24 O.D. (Figure 5a). Only at day 35 was it possible to detect a positivity in all animals. Then, the IgM titres rapidly decreased until they were negative again. The statistical analysis performed does not show any significant difference at any of the compared time points.

Conversely, the immune response against KLH increased in animals that received the vaccine a few days after the injection for IgG antibodies, reaching a first peak O.D. value at day 14 (Figure 5b). A further increase in titres was observed after the booster dose administered at day 28, reaching the highest titre at day 35. The IgG titre was positive until the end of the experiment (day 63). All the IgG values at different time points, except for day 7, were different (*p* < 0.05) when compared against the day 0 value, allowing vaccinated animals to be detected starting from day 14.

### 3.4. Study on Lactation

**Figure 6:**
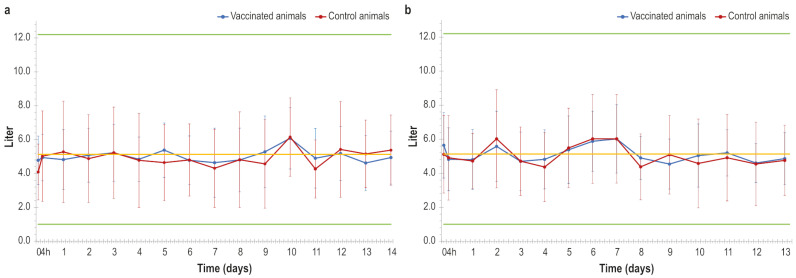
(**a**) Measurements of produced milk in animals (vaccinated and control) after first dose administration, over time. (**b**) Measurements of produced milk in animals (vaccinated and control) after second dose administration, over time. The upper and lower green lines and the yellow line represent, respectively, the highest, lowest and median quantities of milk measured in all the animals before the vaccine trial.

Regarding the quantity of produced milk, the lactating cows that received both doses of the vaccine did not show any statistically relevant differences when compared to the control animals, as shown in Figure 6a,b. Only one animal, belonging to the placebo group, was excluded from the analysis since it was becoming a dry cow. Moreover, about the chemical–physical milk properties, i.e., casein, somatic cells, lipids, proteins and dry fat residue, the statistical analysis did not detect any significant difference between the two groups.

## 4. Discussion

Lumpy skin disease, a transboundary viral disease of cattle and buffalo species, has significant health and economic consequences for the affected countries, such as limitations on trade in animals, slaughter of infected cattle, reduction in milk production, abortion, and infertility of bulls [7,21].

The recent outbreaks in Italy, France, and Spain highlight the urgent need for developing effective preventive measures in order to prevent further LSDV circulation in other European countries.

Prevention tools include surveillance of animals, monitoring of the arthropod vectors and vaccination of susceptible animals.

Vaccination represents the most effective control strategy, as demonstrated by the mass vaccination campaign with a homologous live attenuated vaccine in south-eastern Europe [8], which enabled a successful containment of an LSD outbreak. However, live vaccines pose risks of viral replications causing clinical signs of disease, viral shedding, and safety concerns in LSD-free regions [22,23], which has led to increasing interest in safer alternatives such as inactivated vaccines. Encouraging results have already been reported by Hamdi et al. and Wolff et al. [22,24], who produced inactivated vaccines against LSD, and recently an inactivated vaccine was also produced in our laboratory [15].

This vaccine was previously tested on calves (the age group most susceptible to the LSD) to assess its safety, immunogenicity and efficacy, and in this study we did not perform an efficacy test. In the first study, the vaccine was able to reduce virus replication and clinical signs of disease in vaccinated calves, although it was not a sterilising vaccine. This lack of sterilising immunity suggests that viral shedding may still occur in vaccinated animals, with potential epidemiological implications in terms of virus transmission. Nevertheless, the vaccine substantially reduced the clinical signs of disease during a fatal challenge conducted in calves, in which two control animals were euthanized in order to prevent unnecessary suffering [15]. Although further optimisation is required to improve its efficacy and potentially achieve sterilising immunity, the current formulation was considered suitable for evaluation in lactating animals. Therefore, this vaccine was used in the present study to further investigate its safety and immunogenicity on lactating cows and to exclude its potential side effects on lactation, a critical issue in the dairy industry. Moreover, the vaccine was tested in guinea pigs in order to evaluate this animal species as a preliminary immunogenicity model suitable for further studies on vaccines produced in our laboratory.

Two groups of animals were initially formed, each consisting of ten female Friesian cows: one group was subjected to immunisation with two doses of the above-mentioned vaccine, and the second one was kept as the negative control. Vaccine safety was monitored by measuring the animal temperature daily, performing a general physical examination, and collecting milk samples in the afternoon in order to subsequently analyse potential modifications in milk quantity and chemico-physical properties.

After administration of the two vaccine doses, none of the animals showed adverse reactions, except for one animal that showed a skin nodule at the injection site which spontaneously regressed within a few days. Furthermore, no increase in animal body temperature was detected.

These data suggest a safe profile even better than that reported in our previous study and by Hamdi et al., Wolff et al., and Haegeman et al. [22,24,25], since some fever peaks have been detected in animals after vaccine inoculation in all of these previous studies.

The animals were also subjected to weekly blood sampling in order to assess the vaccine immunogenicity using serological assays.

As expected, no anti-LSDV antibodies were detected in the control group animals, unlike the lactating cows, which showed a strong immune response after 28 days as demonstrated by the results of both the ELISA and SN test. These findings indicate that the second dose administration was needed to elicit an effective immune response in lactating cows, as similarly obtained in our previous vaccine trial on calves [15] and by Haegeman et al. [25].

Otherwise, Hamdi et al. and Wolff et al. [22,24], as shown in their studies, obtained a slightly earlier seroconversion of their animals. In particular, samples were positive already at 21 days after the first vaccine administration. These findings could be related to the viral titre of the vaccine dose before inactivation: their vaccines had viral titres of 10^7^ and 10^6^ TCID_50_/mL, respectively, whereas the titre of our vaccine was 10^5.6^ TCID_50_/mL.

The immune response in guinea pigs was also evaluated in order to explore the possibility of using this animal species as an experimental model for studying the safety and immunogenicity of vaccines against lumpy skin disease in proof-of-concept studies or for potency tests in batch-release studies. In our laboratory, previous experiments have been favourably conducted using guinea pigs as an animal model to assess the safety and immunogenicity of vaccines; in particular, we produced and tested on guinea pigs a vaccine against African horse sickness virus (AHSV) [26,27].

In these studies, both horses and guinea pigs showed a comparable immune response, thus suggesting that guinea pigs could be used as an alternative animal model for testing the antigenic potency of vaccines. Concerning vaccines intended for fighting lumpy skin disease, animal experimentation has been mostly performed on cattle, cows, calves and pregnant heifers [24,28,29,30], but few vaccine studies using guinea pigs as non-ruminant hosts [31] have been reported. The main advantages of the use of rodent models for animal experimentation consist of their small size and the ease of maintenance, thus reducing overall experimental costs [32,33,34].

In our study, it was demonstrated that the guinea pig was a reliable model in which to test the immunogenicity of vaccines intended for lumpy skin disease, since the immune response evaluated in both the ELISA and SN test after immunisation with our vaccine showed similar values between dairy cows and guinea pigs. The immune response in guinea pigs started earlier than that reported in cows, probably due to the amount of vaccine administered being too high (0.5 mL× 2 inoculation in guinea pigs versus 2 mL× 2 inoculation in cows).

Moreover, our study demonstrated that the produced vaccine was able to induce the cell-mediated immune response in lactating cows, which was detected after 7 and 28 days, i.e., after the first and second vaccine dose administrations. These findings confirm that this type of immunological response could be elicited also after immunisation with vaccines different from live attenuated ones, as previously reported by Ronchi et al. [15] and Hamdi et al. [22] for inactivated vaccines against LSD, and Gu et al. [35] and Wen et al. [36] for new generation LSD vaccines, in addition to results reported for inactivated vaccines against other viruses [37,38,39].

Furthermore, in this study the results about the potential use of the exogenous protein KLH as a positive marker for vaccinated animal identification in the eradication campaign were confirmed, as stated in our previous findings [15]. The use of the KLH as a positive marker without a negative marker (deletion) in the vaccine limits the full application of the DIVA strategy for our formulation. The KLH included into our vaccine formulation allows for discrimination between vaccinated and naturally infected animals, since KLH is an exogenous protein derived from *Megathura crenulata*, thus antibodies against KLH are exclusively induced by vaccination and are absent in naturally infected animals. A potential limitation is represented by the identification of vaccinated animals that could be infected, which may not be distinguishable with this approach. Nevertheless, this strategy could help in a vaccination plan where the use of a unique vaccine is mandatory.

The results of a KLH ELISA performed on lactating cow serum samples showed a strong antibody response only for IgG-KLH, in contrary to IgM antibodies which showed a weak positivity only one week after the second vaccine dose administration. In our previous experiment, where we performed the challenge on calves, the KLH antibody titre was monitored, and the positivity was also confirmed during the challenge. Although a challenge was not performed in the present study, these findings support the consistency of KLH as a serological marker of vaccination. In particular, the trend of IgG titre values obtained on calves was similar to the trend recorded in lactating cows. On the contrary, the IgM titres obtained in our previous experiment, with O.D. values reaching two peaks in two different animals (2.96–3.15), appear to be higher than the titre values obtained in lactating cows, with a single animal peak of 1.24. That last finding needs to be investigated deeper in order to evaluate if parameters like age and/or sex could affect the KLH-IgM immune response elicited by our DIVA vaccine.

Therefore, although a similar KLH IgG immunogenicity pattern between vaccinated calves and lactating cows has been pointed out, more studies should be conducted on lactating cows in order to confirm the encouraging results about the application of the positive marker strategy in this vaccine.

Finally, yet importantly, our study evaluated the effects of this vaccine formulation on milk production in dairy cows at different stages of lactation.

In particular, milk production was monitored analysing both yield and chemical–physical parameters (milk volume, fat content, protein, lactose, casein, dry residue and somatic cell count). The results showed that the administration of our vaccine did not negatively affect either the quantity or the quality of the milk produced, in contrast to the milk reduction detected in naturally infected herds as well as in animals treated with live attenuated vaccines [12,40,41].

The absence of statistically significant differences in milk production between the vaccinated and control group underlines the safety of our vaccine in production settings.

Moreover, the lack of any adverse reactions or effects on milk production in cows, together with the safety and immunogenicity properties, supports the hypothesis that following further optimisation aimed at improving its efficacy this vaccine may represent a suitable candidate for potential field application without adversely affecting milk production. This is supported by the findings of the present study, which demonstrated the absence of measurable effects on milk yield and chemico-physical parameters.

## 5. Conclusions

The present study aimed to assess the safety and immunogenicity of an inactivated vaccine in a guinea pig animal model and in lactating cows. In this last species the effect of the vaccine on milk production in both qualitative and quantitative terms was evaluated. The obtained results demonstrated that the vaccine was characterised by a satisfactory safety profile, since any systemic side effects were not observed, and by a high immunogenicity response in both guinea pigs and lactating cows.

Moreover, the vaccinated lactating cows kept stability in milk production, suggesting that vaccination did not negatively interfere with lactation performance. This represents an aspect particularly relevant for milk producers, for which the maintenance of productivity is a key requirement. The use of this type of vaccine could ensure the application of vaccination programmes without disrupting farm productivity, since this vaccine is immunogenic and also limits clinical signs of the disease. In addition, these results highlight the potential usefulness of guinea pigs as a safety and immunogenicity animal model in LSD vaccine development processes. Furthermore, this vaccine could represent a safe and immunogenic tool in LSD vaccination campaigns, allowing the identification of vaccinated animals. However, further studies should be conducted in order to define the vaccine performance in lactating cows after an experimental challenge, which was not performed in the current study.

In conclusion, although this inactivated vaccine would benefit from further improvements to enhance its efficacy and to extend its application within a DIVA strategy (with negative marker), it could be a safer alternative to live attenuated vaccines in the control of LSD in dairy cows because it did not affect the milk production, which is one of the most critical issue in the dairy farm industry.

## Data Availability

All data acquired are shown in the paper.

## Abbreviations

The following abbreviations are used in this manuscript:

LSD	Lumpy skin disease
DIVA	Differentiating Infected from Vaccinated Animals
MDBK	Madin–Darby bovine kidney
KLH	Keyhole limpet hemocyanin
EURL	European Union Reference Laboratory
OBP	Onderstepoort Biological Products
LSDV	Lumpy skin disease virus
OA3	Ovine Aries Testis cell line
TMP	Trans-Membrane Pressure
MEM	Minimum Essential Medium
BEI	Binary Ethylenimine
PBS	phosphate-buffered saline
ELISA	enzyme-linked immunosorbent assay
IFN-γ	interferon-γ
SN	Serum neutralisation
TCID_50_	Tissue Culture Infectious Dose 50%
AHSV	African horse sickness virus
